# The Rates of Protein Synthesis and Degradation Account for the Differential Response of Neurons to Spaced and Massed Training Protocols

**DOI:** 10.1371/journal.pcbi.1002324

**Published:** 2011-12-29

**Authors:** Faisal Naqib, Carole A. Farah, Christopher C. Pack, Wayne S. Sossin

**Affiliations:** 1 Department of Physiology, Montreal Neurological Institute, McGill University, Montreal, Quebec, Canada; 2 Department of Neurology and Neurosurgery, Montreal Neurological Institute, McGill University, Montreal, Quebec, Canada; North Carolina State University, United States of America

## Abstract

The sensory-motor neuron synapse of *Aplysia* is an excellent model system for investigating the biochemical changes underlying memory formation. In this system, training that is separated by rest periods (spaced training) leads to persistent changes in synaptic strength that depend on biochemical pathways that are different from those that occur when the training lacks rest periods (massed training). Recently, we have shown that in isolated sensory neurons, applications of serotonin, the neurotransmitter implicated in inducing these synaptic changes during memory formation, lead to desensitization of the PKC Apl II response, in a manner that depends on the method of application (spaced versus massed). Here, we develop a mathematical model of this response in order to gain insight into how neurons sense these different training protocols. The model was developed incrementally, and each component was experimentally validated, leading to two novel findings: First, the increased desensitization due to PKA-mediated heterologous desensitization is coupled to a faster recovery than the homologous desensitization that occurs in the absence of PKA activity. Second, the model suggests that increased spacing leads to greater desensitization due to the short half-life of a hypothetical protein, whose production prevents homologous desensitization. Thus, we predict that the effects of differential spacing are largely driven by the rates of production and degradation of proteins. This prediction suggests a powerful mechanism by which information about time is incorporated into neuronal processing.

## Author Summary

Memories are among an individual's most cherished possessions. One factor that has been shown to exert a powerful influence on memory formation is the pattern of training. Learning trials distributed over time have been shown to consistently produce longer lasting memories than trials distributed over short intervals, in every organism in which this has been studied. This observation has been investigated particularly well in the marine mollusk *Aplysia californica*. The nervous system of *Aplysia* is simple and well characterized, yet capable of forming memories, making it an ideal system for the study of learning and memory. Currently, we have a detailed understanding of memory formation in *Aplysia* at the cellular level. However, there remain many unanswered questions at the molecular level, particularly concerning how the effects of different patterns of learning are mediated. We have developed a mathematical model of a molecular signaling pathway known to underlie memory formation in *Aplysia*. Our model suggests that the rates of synthesis and degradation of proteins involved in memory regulation are essential for neurons of *Aplysia* to respond differentially to spaced and massed training. We were able to experimentally validate these findings, thus providing significant evidence for this model, which might underlie memory formation in more complex animals.

## Introduction

Different patterns of training can lead to different types and strengths of memories. For example, training distributed over time (spaced training) is superior to the equivalent amount of training with no interruptions (massed training) for generating long-term memories for verbal tasks [1]. Spaced and massed training are known to activate different molecular signaling pathways underlying memory formation [2]. *Aplysia californica*, a marine mollusk, provides an ideal model system for examining the differences in molecular signaling mediated by spaced and massed training [3].

One form of behavioral sensitization in *Aplysia* involves an increase in defensive reflexes after a noxious stimulus. The increase in defensive reflexes is caused in part by an increase, or facilitation, of the strength of the synapse between the mechanoreceptor sensory neurons and withdrawal motor neurons [4]. Facilitation is mediated by release of serotonin (5HT) from interneurons activated by the noxious stimulus [5], [6]. Spaced noxious stimuli are superior to massed stimuli at generating long-term sensitization in the animal [3] and spaced applications of 5HT are superior to massed applications at generating long-term facilitation (LTF) of cultured sensory-motor neuron synapses [7]. The ability to examine the difference between spaced and massed training in cultured neurons allows the study of the differential signaling events activated by spaced and massed training.

**Figure 1:**
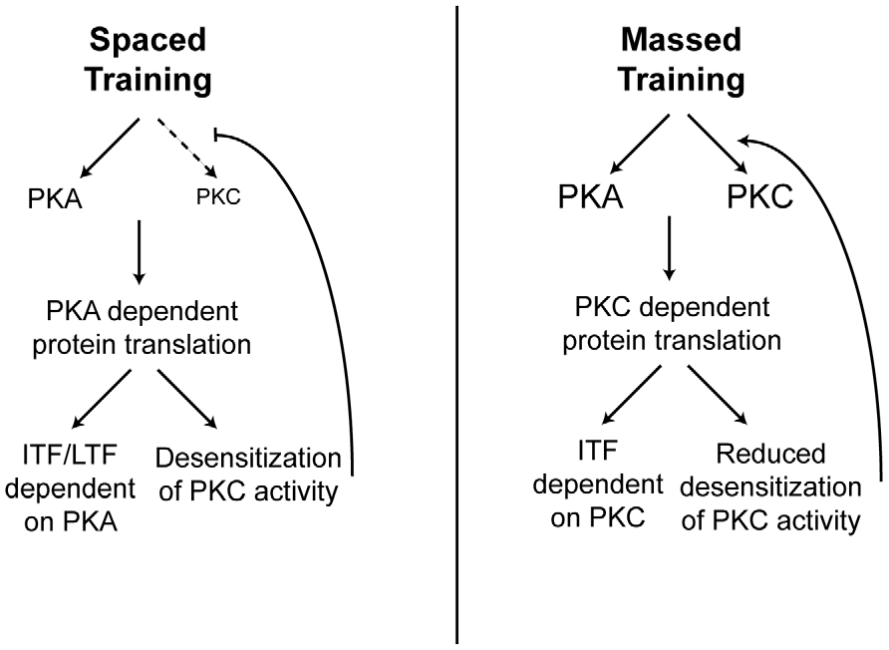
Massed versus spaced application of 5HT. Spaced training activates PKA but not PKC and leads to PKA-dependent translation that induces intermediate-term facilitation (ITF) and long-term facilitation (LTF) not dependent on PKC. PKA dependent translation also produces a protein that increases PKC desensitization, which is required for spaced training not to activate PKC. Massed training activates both PKA and PKC and leads to PKC-dependent translation that induces a distinct form of ITF not dependent on PKA. PKC-dependent translation also produces a protein that prevents PKC desensitization, which is required for massed training to continually activate PKC.

5HT acts through at least two distinct G protein coupled receptors (GPCRs) in *Aplysia* to activate protein kinase A and protein kinase C [8], [9]. The two kinases are differentially activated based on the type of training; spaced applications of 5HT lead to the persistent activation of PKA in the sensory neuron [10], [11], while massed applications of 5HT instead activate both PKA and the novel PKC Apl II in the sensory neuron (Figure 1) [10], [12].

An important mechanism for the differential activation of PKC during spaced and massed applications of 5HT involves differential desensitization of PKC Apl II translocation to the plasma membrane, where it is activated [13]. Spaced training (5×5 min 5HT with 15 min wash periods in between) leads to more desensitization than one massed 25 min application of 5HT [13]. This differential desensitization is surprising, since spaced applications of 5HT allow the neuron to recover in between exposures; yet they cause a greatly increased amount of desensitization when compared to the massed application of 5HT. This effect was shown to depend on both PKA-mediated desensitization and the downstream effects of protein synthesis [13]. Importantly, protein synthesis inhibitors have opposite effects depending on the training stimulus: massed training produces a protein that prevents desensitization of PKC Apl II translocation while spaced training produces a protein that promotes desensitization of PKC Apl II translocation (Figure 1) [13]. Thus, another important distinction between these two training paradigms is that they activate distinct translational pathways.

While massed applications of 5HT are less effective than spaced applications at generating LTF measured at 24 h [7], both spaced and massed training lead to protein-synthesis dependent intermediate-term facilitation (ITF), measured 30 min to 2 hr after 5HT is removed [11], [14], [15]. However, the mechanisms underlying ITF induced by spaced or massed training are distinct; ITF induced by spaced training require PKA but not PKC for induction [16], [17], while ITF induced by massed training, even a continuous stimulus as short as 10 min, requires PKC but not PKA [14] (Figure 1). Thus, the differential activation of PKC during massed and spaced training appears critical for the different physiological effects of these two training paradigms.

In order to better understand the signaling pathway mediating the desensitization of PKC Apl II, we developed a model consisting of a system of integro-differential equations describing the differential desensitization of PKC Apl II activation during massed and spaced training. The model provides predictions about the molecular mechanisms responsible for the differences between massed and spaced training. These predictions were validated with new experiments. Together these results suggest that the sensitivity of neurons to the time between training periods is due to the rates of protein synthesis and degradation.

## Results

### Describing the model architecture

We have previously described PKC Apl II translocation and its desensitization in response to 5HT application in the presence of PKA and protein synthesis inhibitors [13], [18], [19]. We showed that PKC translocation differentially desensitizes to spaced and massed applications of 5HT, and that this differential desensitization was dependent on protein translation and PKA activity. In order to understand the molecular mechanisms underlying desensitization of PKC Apl II translocation we designed a signaling network based on our previous experimental findings and biochemical mechanisms known to underlie G protein-coupled receptor (GPCR) desensitization. Our network consists of the translocation of PKC, the cycling of a GPCR, the translation of two hypothetical proteins, and activity of PKA. We have tried to simplify the network whenever possible, including bundling multiple biochemical reactions into one single rate in order to simplify its architecture. The reasoning behind the network's architecture is given in this section and the model equations are given in the Materials and Methods section.

The basic unit of the model is the 5HT GPCR (S) that once activated leads to the production of diacylglycerol (DAG), which is capable of activating and translocating PKC Apl II to the membrane [20], [21]. While this pathway consists of multiple steps, such as G-protein activation of phospholipase C and phospholipase D [18], these are not likely to be important for modeling of desensitization, since in most systems the amount of the activatable GPCR is the rate-limiting quantity that is decreased during desensitization [22], [23], [24].

GPCRs can enter a number of different pathways, such that S can exist in several different states, where the change in concentration of each state with respect to time is modeled. The base component of our model includes the activation and inactivation of S without any desensitization dynamics. This component corresponds to how quickly PKC Apl II translocates to the membrane after 5HT application and how quickly it dissociates from the membrane after 5HT is washed away. It is known that application of 5HT results in a maximal translocation of PKC Apl II within one minute, after which it remains at this maximal level for at least five minutes [18], [19]. Washing off 5HT prompts the complete dissociation of PKC Apl II within one minute [13], [18], [25]. To replicate these findings, we used a simple network architecture, whereby in the presence of 5HT, S_OFF_ becomes S_ON_, which then transforms to S_IN1_. S_OFF_ represents the inactivated receptor that can become activated by 5HT, turning S_OFF_ into S_ON_, which then produces DAG allowing for the translocation of PKC Apl II. S_IN1_ is an inactivated receptor that needs to be recycled before it can become activated by 5HT again. At a biochemical level, the transitions from S_ON_ to S_IN1_ to S_OFF_ involve multiple molecular steps including GPCR phosphorylation by G protein receptor kinases, binding of beta arrestin, possible internalization of the receptor, unbinding of the ligand, and then recycling of the receptor back to its initial state [22], [23], [24]. For simplicity, we have reduced these multiple steps into the two steps (S_ON_ to S_IN1_ to S_OFF_) since (i) this is sufficient to capture the behavior required to understand the questions we are addressing (see below) and (ii) we have no specific knowledge concerning regulation of these pathways in *Aplysia*. The major constraint from the data is that PKC comes off the membrane in less than one minute after 5HT is washed off. Thus S_ON_ to S_IN1_ must be fast enough to account for this inactivation. However, in the first 5 min of 5HT activation, there is little desensitization of PKC Apl II translocation. Thus, S_IN1_ to S_OFF_ must be rapid enough to prevent appreciable desensitization in the first five minutes. The transitions between states of S were modeled using mass action kinetics. These model parameters were fit to the previously described PKC dynamics [13], [18], [25] (equations, parameter values, and parameter estimation methods can be found in the Materials and Methods section). Once an appropriate fit was found these parameters were set and we were able to begin expanding the model and modeling data related to PKC Apl II desensitization.

**Figure 2:**
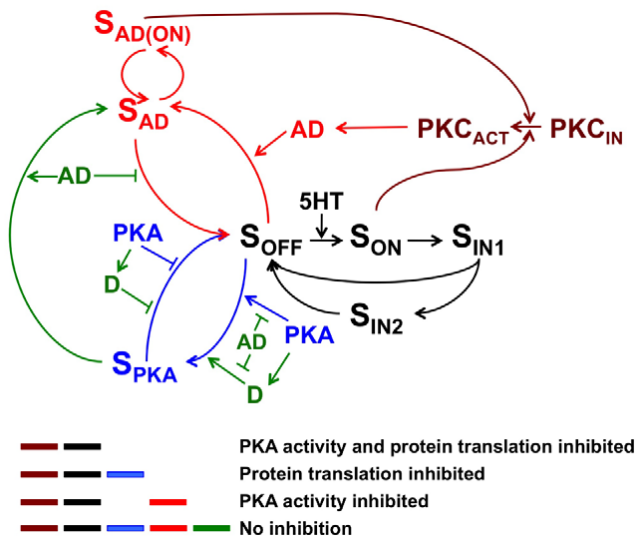
Complete model network. The maroon network describes the translocation of PKC to the plasma membrane and its subsequent dissociation from the membrane. The module denoted in black represents the homologous desensitization pathway. The blue network defines the PKA mediated desensitization of PKC Apl II. The red network illustrates the AD pathway responsible for the rescuing PKC from desensitization. Finally the D pathway, which is antagonistic to the AD pathway and causes the increase in desensitization, is specified by the green network. In green are the additional roles of AD needed to counteract D during massed training.

The complete model architecture is presented in Figure 2. The model components (color coded) were developed sequentially, with maroon and black first then blue, red, and finally green. The maroon component represents only the translocation of PKC to the plasma membrane and its subsequent dissociation from the membrane. The black component represents the desensitization pathway in the presence of a protein translation inhibitor and a PKA inhibitor. In the presence of these inhibitors, PKC Apl II translocation desensitizes during exposure to 5HT [13]. Thus, there must be a protein translation-independent and PKA-independent desensitization pathway, or a homologous desensitization pathway, which we model as an alternate recycling pathway from S_IN1_ to S_OFF_, passing through S_IN2_ (Figure 2; black network only, equations can be found in the Materials and Methods section). Here S_IN2_ acts as a secondary inactivated state that requires a longer processing time than S_IN1_ before recycling back to S_OFF_. At the biochemical level, this represents the sorting of the GPCR in the endocytic compartment from a rapid recycling pathway into a slow recycling pathway or degradative pathway. This architecture was chosen because of the abundant literature supporting this mechanism for desensitization of GPCRs [22], [23], [24].

PKA, which is activated by 5HT, has been shown to increase desensitization of PKC Apl II translocation in the absence of protein translation [13]. The condition where PKA is active and protein translation is inhibited is modeled by the combination of the black, maroon, and blue components. In order to model PKA-mediated protein synthesis-independent desensitization, we included a reduced and modified version of a previous model of PKA activity [26]. Our modifications to this PKA model are described in the next sections. Activity of PKA is capable of converting S_OFF_ directly into S_PKA_, where S_ON_ is not immediately attainable and PKC Apl II cannot be activated (Figure 2; black and blue networks, equations can be found in the Materials and Methods section). At the biochemical level, this network would represent phosphorylation of the receptor, or receptor-associated protein, by PKA causing the endocytosis of the GPCR from the plasma membrane to an endocytic compartment distinct from S_IN1_ and S_IN2_, probably representing a regulated recycling endosome [27]. It is important to note that since PKA can convert S_OFF_ to S_PKA_, conversion to S_PKA_ does not require S to go through the active state, S_ON_, such as the desensitization mediated by S_IN2_. This network architecture is required to account for the observation that PKA activity between pulses of 5HT, when S would not be activated, is capable of desensitizing PKC Apl II translocation [13] and is consistent with data on heterologous desensitization of GPCRs in other systems [28]. This consideration also removed the alternate topology where S_PKA_ would represent alternate sorting from S_IN1_, since the receptor is only in the S_IN1_ state when the receptor goes through the active state.

The recycling of S_PKA_ back into S_OFF_ is inhibited by PKA. This inhibition was not initially part of the architecture, but it was not possible to replicate both the massed training and spaced training data sets without including the PKA inhibition of S_PKA_ recycling (see results below). At a biochemical level, this suggests that PKA activity is not only required to induce sorting of the receptor to the regulated recycling endosome but its retention in this compartment as well.

The reverse situation, with PKA activity inhibited but protein translation allowed to function is modeled by the combination of the black, maroon, and red components. Protein translation in the absence of PKA activity leads to a reduction in the desensitization of PKC Apl II translocation only during massed 5HT application and not spaced [13]. This observation requires that a protein, which protects PKC Apl II translocation from the constitutive desensitization pathway be translated during massed training. We name this hypothetical protein Anti-Desensitizer (AD) and its effects on the network are represented by the black, maroon and red components combined. We modeled the mechanism of AD mediating this protection by having AD convert S_OFF_ into S_AD_, a form of S preserved from the desensitization pathways leading to S_IN2_ or S_PKA_, but similar to S_OFF_ in its ability to become activated by 5HT and cause the translocation of PKC Apl II (Figure 2, black, maroon and red pathway; equations can be found in the Materials and Methods section). At the biochemical level, this would represent the AD protein binding to the receptor, or receptor associated protein, preventing its inactivation and internalization [29], [30], [31], [32]. Since a protein-synthesis dependent protection from desensitization is seen in massed, but not spaced, training protocols, we would expect AD to be synthesized only after massed training. In order for this differential synthesis to occur, we made production of AD proportional to the mathematical integration of the level of active PKC Apl II. PKC Apl II is constantly active during massed training, but not during spaced training; thus, integrating PKC activity allows for selective activation of AD during massed training. PKC is known to regulate the translational machinery in many systems [33], [34] including *Aplysia* [35], [36], but the exact mechanism by which PKC regulates translation in this case is not known and is not explicitly modeled here.

Finally, allowing both protein translation and PKA activity to proceed normally results in an increase in the desensitization of PKC Apl II translocation during spaced training [13]. This increase in desensitization was observable only when both PKA activity and protein translation are allowed to proceed, meaning a translated protein is mediating this increase in desensitization, and its rate of translation is dependent on PKA activity. We name this hypothetical protein Desensitizer (D), and we model its mechanism of action similarly to that of PKA by transforming S_OFF_ into S_PKA_ and inhibiting its recycling back to S_OFF_ (Figure 2, complete network; equations can be found in the Materials and Methods section). Another possible architecture would have been to generate another state of S (S_D_), but there was not a good biochemical rationale for this and the model worked well (see below) without this additional state. At the biochemical level, D would be a protein that promotes endocytosis [29], particularly to the PKA-dependent pathway. The rate of translation of D is dependent on the amount of PKA activity, similar to the dependence of AD translation on PKC Apl II activity. One difference between the translation of D and AD is that D's production is delayed by 10 min after its induction. The use of a delay was necessary to account for the observation that desensitization of PKC Apl II translocation after a 5 min pulse of 5HT did not begin until after a 10 min wash [13]. At a biochemical level, there may be many reasons for a delay, ranging from requirements for post-translational modification, cellular trafficking, or delay in the activation of proteins synthesis. Finally, while trying to model the data we found that for D to cause enough desensitization during spaced training resulted in too powerful an inhibition during massed training. This over-inhibition resulted from the fact that unlike AD, D is synthesized during both spaced and massed training since PKA is active in both scenarios [10]. To diminish the role of D during massed training, we introduced two additional effects of the AD protein. First, AD inhibited the transition from S_OFF_ to S_PKA_, and second, it could transform not only S_OFF_ to S_AD_ but also S_PKA_ to S_AD_ (Figure 2; complete network). At a biochemical level, this corresponds to the ability of the AD protein to prevent endocytosis to the PKA-dependent pathway, and moreover, to bind to the GPCR in the regulated recycling endosome and enhance its recycling, similar to the mechanism by which decreased PKA activity enhanced recycling from this compartment. We also attempted to model the system with AD preventing the translation of D as opposed to opposing its actions, but were unable to achieve a good fit to the data with this architecture.

For simplicity, we made the assumption that during the time course of our experiments an insignificant amount of new S is created. This assumption was also made partially because for S to enter the S_OFF_ state, the GPCR would not only have to be synthesized, but processed through the endoplasmic reticulum, Golgi apparatus, and transported back to the membrane, so new S could only contribute to the later parts of the experimental paradigm. We do not have a term for destruction of S, however, as described below, the S_IN2_ pathway may be equivalent to a degradation pathway, where the GPCR enters late endosomes and lysosomes.

### Modeling the homologous desensitization pathway finds slow rate of recovery from desensitization

**Figure 3:**
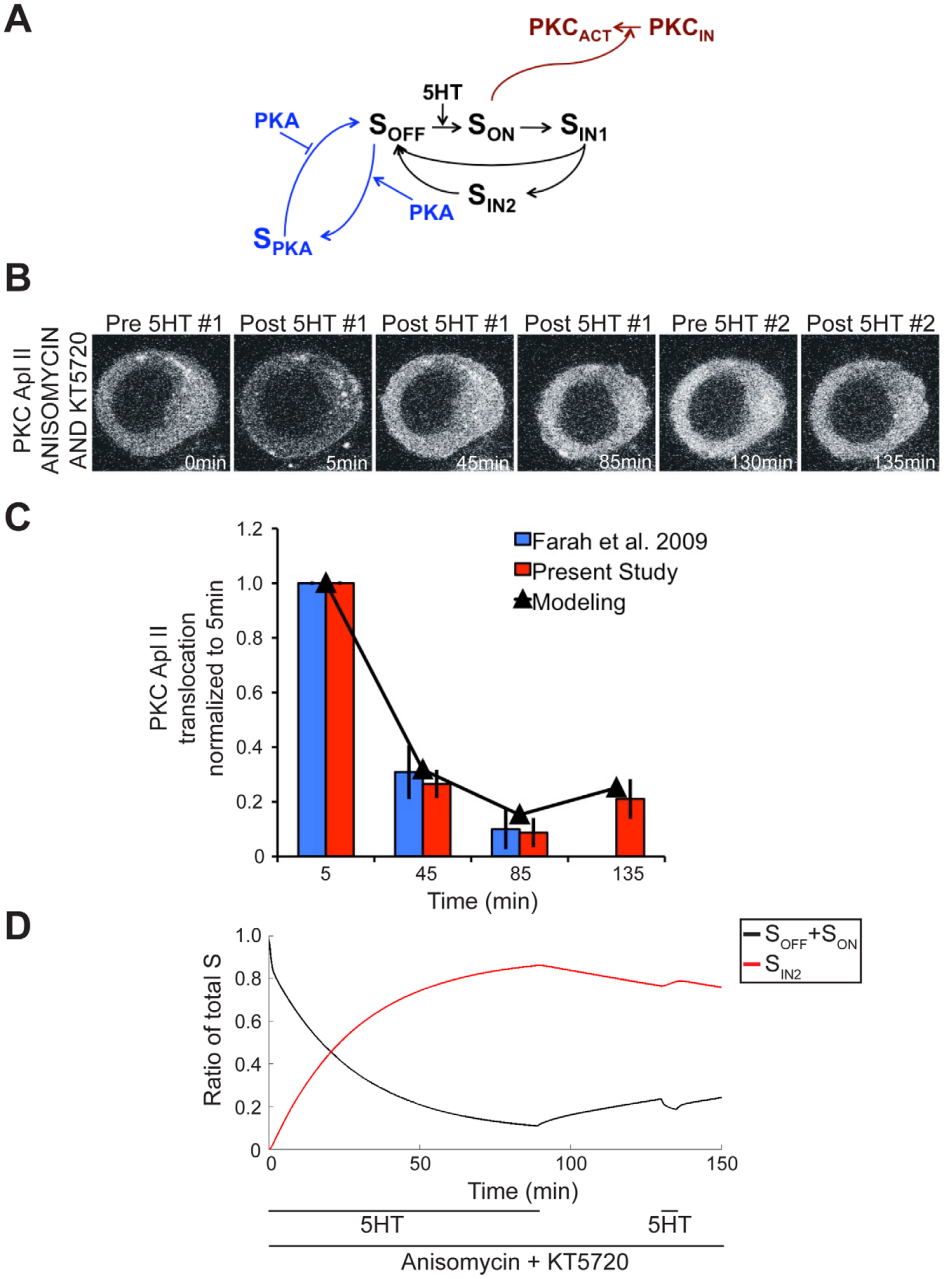
Modeling and experimental validation of homologous desensitization pathway. **A**, Model network pathways of homologous desensitization pathway (black) and PKA-mediated desensitization pathway (black and blue). **B**, Representative confocal fluorescence images of sensory neurons expressing eGFP-PKC Apl II during a 90 min exposure to 5HT followed by a 45 min wash and then a 5 min 5HT application, all in the presence of anisomycin and KT5720. **C**, Quantification of PKC Apl II translocation (bars) and modeling output (line). Blue bars are data used from Farah et al. (2009) to fit the model parameters. Red bars are data from the present study (n = 8 cells). Error bars are SEM. **D**, Modeling of S dynamics in response to experimental protocol from **B**. Black line represents the ratio of S_OFF_ and S_ON_ to total S and the red line the ratio of S_IN2_ to total S. The times of addition of 5HT and pharmacological agents are indicated below the figure.

PKC Apl II translocation still desensitizes during exposure to 5HT even when both protein translation and PKA have been inhibited [13]. Thus, there must be a homologous desensitization pathway (Figure 3A; black network only, equations can be found in the Materials and Methods section). Parameter values were estimated by fitting the model to PKC Apl II translocation measurements taken during a continuous 90 min application of 5HT in the presence of the protein translation inhibitor anisomycin and the PKA inhibitor KT5720 [13]. Several parameter estimation methods were used, and surprisingly, all of them yielded recycling rates of S_IN2_ back to S_OFF_ (k_A5_) that were near zero (parameter values can be found in Table 1), resulting in an excellent fit to the data as can be seen in Figure 3C (R^2^>0.99). Note that throughout the paper, data presented in blue represents data obtained from Farah et al. (2009) used to train the model, while data presented in red represents experiments performed to confirm predictions of the model. The model predicted very little recycling of the signaling complex from S_IN2_ during massed training in the absence of protein translation and PKA activity (Figure 3C, D). This was unexpected, since our earlier experiments showed that the desensitization seen after a 5 min pulse of 5HT recovered completely within 45 min, suggesting efficient recycling of the signaling complex [13]. However, these experiments were not done in the presence of a PKA inhibitor.

**Table 1:** Model parameters.

Parameter	Value	Sensitivity (Spaced)	Sensitivity (Massed)	Notes (Found in Equation(s))
k_A2_*	1	High	High	S_ON_ into S_IN1_ (2,4)
k_S1a_	6	High	High	Half saturation of Hill function synthesizing AD (10)
k_D3b_	0.3372	High	High	Hill coefficient of Hill function inhibiting S_OFF_ into S_PKA_ (via AD) (3,6)
k_DAGp_*	200	High	High	DAG synthesis rate constant (1)
k_DAGd_*	10^2^	High	High	DAG degradation rate constant (1)
k_A1_*	10^5^	High	Medium	S_OFF_ into S_ON_ (2,3)
k_S1b_	4	High	Medium	Hill coefficient of Hill function synthesizing AD (11)
k_S3_	0.4483	High	Medium	D synthesis rate constant (13)
k_A3_*	3	Medium	High	S_IN1_ to S_OFF_ (3,4)
k_A4_*	0.2371	Medium	High	S_IN1_ to S_IN2_ (4,5)
k_S3a_	6	Medium	High	Half saturation of Hill function synthesizing D (13)
delayD	10	Medium	High	PKA delay in D synthesis (14)
intPKA	15	Medium	High	PKA integration window (14)
k_D3_	0.0764	Medium	Medium	S_PKA_ into S_AD_ (6,15)
k_D3b_	0.1385	Medium	Medium	Hill coefficient of Hill function activating S_PKA_ into S_AD_ (6,15)
k_S1_	0.026	Medium	Medium	AD synthesis rate constant (11)
k_S2_	0.2	Medium	Medium	AD degradation rate constant (12)
k_C1_	2	Medium	Medium	S_OFF_ into S_AD_ (3,15)
k_C2_	0.1	Medium	Medium	S_AD_ into S_OFF_ (3,15)
k_S4_	0.2847	Medium	Medium	D degradation rate constant (13)
k_D1_	8.0441	Medium	Medium	S_OFF_ into S_PKA_ (via D) (3,6)
k_D1a_	5.33*10^−8^	Medium	Medium	Half saturation of Hill function inhibiting S_OFF_ into S_PKA_ (via AD) (3,6)
V_m_	3.6	Medium	Medium	cAMP synthesis rate constant (7)
K_fpka_	105	Medium	Medium	PKA subunit dissociation rate constant (8,9,10)
intPKC	15	Medium	Medium	PKC integration window (12)
k_A5_	0.003	Low	Medium	S_IN2_ to S_OFF_ (3,5)
k_B2a_	0.5	Low	Medium	Half saturation of Hill function inhibiting S_PKA_ into S_OFF_ (via PKA) (3,6)
k_B2b_	6	Low	Medium	Hill coefficient of Hill function inhibiting S_PKA_ into S_OFF_ (via PKA) (3,6)
k_C2b_	1	Low	Medium	Hill coefficient of Hill function inhibiting S_AD_ into S_OFF_ (3,15)
k_B2_	0.2	Medium	Low	S_PKA_ into S_OFF_ (3,6)
k_D2b_	0.4187	Medium	Low	Hill coefficient of Hill function inhibiting S_PKA_ into S_OFF_ (via D) (3,6)
K5HT	14*10^−6^	Medium	Low	Half saturation of Hill function synthesizing cAMP (7)
K_bpka_	3	Medium	Low	PKA subunit reassociation rate constant (8,9,10)
k_B1_	0.1276	Low	Low	S_OFF_ into S_PKA_ (via PKA) (3,6)
k_D3a_	1.6*10^4^	Low	Low	Half saturation of Hill function transforming S_PKA_ into S_AD_ (6,15)
k_C2a_	1	Low	Low	Half saturation of Hill function inhibiting S_AD_ into S_OFF_ (3,15)
k_B2a_	53.1	Low	Low	Half saturation of Hill function inhibiting S_PKA_ into S_OFF_ (via D) (3,6)
cAMP_basal_	0.005	Low	Low	Basal concentration of cAMP (7)
k_S3b_	4	Low	Low	Hill coefficient of Hill function synthesizing D (13)

Parameter sensitivity analysis. Model parameters and their values. Sensitivity was determined by varying individual parameters by +/−5% and +/−50% while holding the other parameters at their defined values. The sensitivity of a parameter was classified as High if either a +/−5% change in its value caused a change in the fit of the data of over 25%. Similarly, the sensitivity of a parameter was Medium if either a +/−50% change in value caused a change in the fit of the data of over 25%, and Low if the +/−50% change in value did not change the fit by more than 25%. Two data sets were used to conduct this analysis: 90 min 5HT and 5×5 min 5HT with 15 min washes. List ordered by sensitivity to 5×5 min 5HT. Parameters indicted with a * were replaced with the following in an alternate model: k_A2_ = 2, k_DAGp_ = 2, k_DAGd_ = 200, k_A1_ = 200,000, k_A3_ = 2, and k_A4_ = 0.08.

To test the prediction of the model that desensitization seen in the absence of PKA activity was not reversible, we conducted a new experiment. The rate of S_IN2_ recycling was predicted to be slow enough that a wash period after massed training with anisomycin and KT5720 would result in little recovery of translocation to initial values. Thus, in a simulation of a 90 min exposure to 5HT followed by a 45 min wash and then a 5 min pulse of 5HT, all in the presence of anisomycin and KT5720, the 5 min pulse of 5HT should only cause a small amount of PKC Apl II translocation, since a majority of S is held in the inactivated state S_IN2_ (Figure 3C, D). To test this prediction of the model, we used this protocol in a new imaging experiment using *Aplysia* sensory neurons expressing eGFP-PKC Apl II. The initial massed training caused a similar amount of translocation to that previously observed by Farah et al. (2009) (Figure 3B, C). Furthermore, the amount of desensitization after the 5 min pulse of 5HT matched the modeling prediction extremely well, demonstrating that recovery from desensitization under these conditions was indeed very slow (Figure 3B, C).

This protocol required that the neurons be imaged for a total of 140 min. To ensure that the lengthy exposure to room temperature (20–23°C) and the drugs anisomycin and KT5720 had no effect on the health of the neurons, or their ability to translocate PKC Apl II, two 5 min pulses of 5HT were applied with a 130 min wash in between, all in the presence of both drugs. Recovery from a 5 min pulse of 5HT occurs after 45 min [13], so we expect that a 130 min wash should result in complete recovery and that any depression in PKC Apl II translocation would be caused by injury to the neurons due to prolonged exposure to room temperature and drugs. There was no significant difference in the amount of PKC Apl II translocation between the first and second pulse of 5HT (mean+/−sem; 1.08+/−0.18, n = 5). Thus the persistent desensitization observed in the previous experiment is due only to accumulation of S in S_IN2_, as predicted by the model and not due to injury to the neurons.

### Modeling desensitization induced by PKA confirms rapid rate of recovery

**Figure 4:**
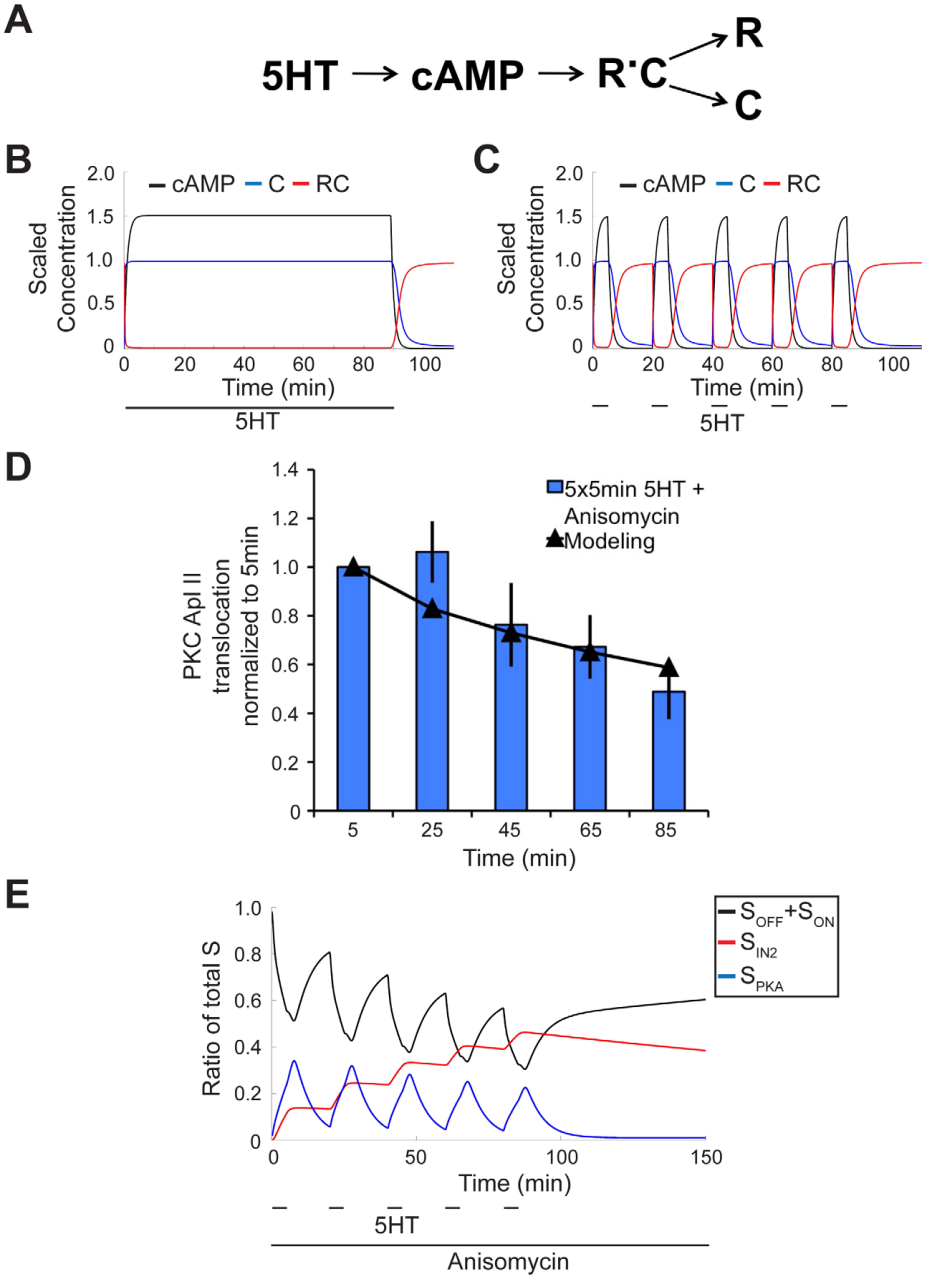
PKA dynamics. **A**, Model network pathway of PKA dynamics. R and C represent the regulatory subunit and catalytic subunit of PKA, respectively, where the amount of PKA activity is considered identical to C activity. **B**, **C**, PKA activity in response to a 90 min 5HT application (**B**) or 5×5 min 5HT application (**C**). Black line represents the amount of cAMP activity; blue line represents C, and red line RC. R is not shown, as it is identical to C. (Model adapted from [70]). **D**, PKC Apl II translocation in response to 5×5 min application of 5HT with 15 min washes in between and anisomycin present throughout from Farah et al. (2009) (bars) and modeling output (line). **E**, Modeling of S dynamics in response to experimental protocol from **D**. Black line represents the ratio of S_OFF_ and S_ON_ to total S, the red line the ratio of S_IN2_ to total S, and the blue line represents ratio of S_PKA_ to total S.

PKA, which is activated by 5HT, has been shown to increase desensitization of PKC Apl II translocation during both massed and spaced training [13]. In order to model PKA mediated desensitization, we included a reduced and modified version of a previous model of PKA activity [26]. We reduced the complexity of this model to only include only the dynamics of cAMP production and the association and dissociation of the subunits of PKA. This simplification was done since our experiments and simulations do not occur over long enough time periods for us to expect a contribution from the persistent activity of PKA, which was a major feature of their model. We modified the Pettigrew et al. model by altering the basal level of cAMP and the association rate of the PKA subunits to refine PKA dynamics to better match published data demonstrating PKA activity persisting for a small period after washout of 5HT [10], [37], [38]. This revision was necessary since PKA activity during the wash period is required for desensitization [13]. The new PKA dynamics to massed and spaced training can be seen in Figure 4A–C. Furthermore, we removed any synthesis or degradation of PKA subunits since, similar to PKC Apl II, we do not expect a significant change in the amount of protein during the time course of our experiments [10].

**Figure 5:**
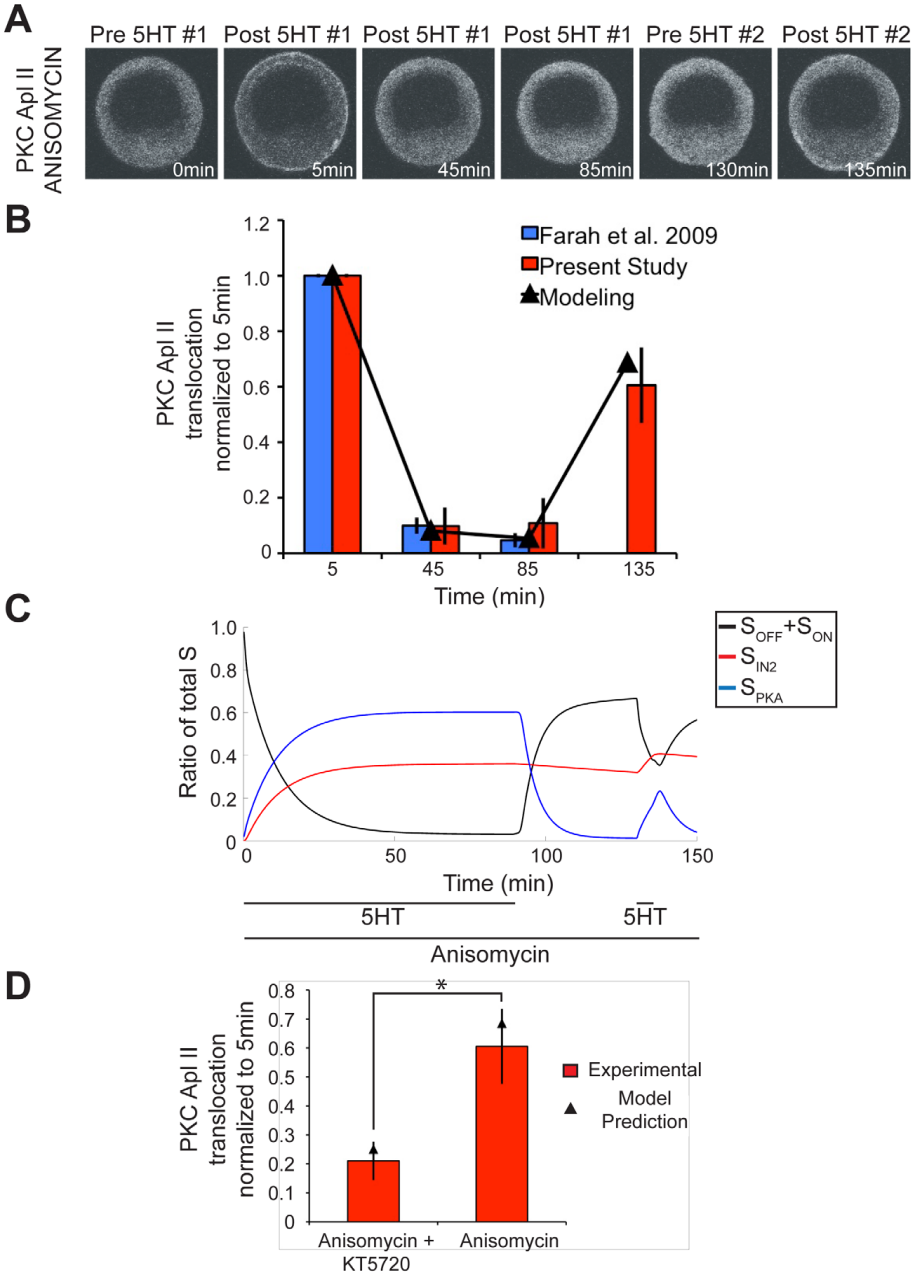
Modeling and experimental validation of desensitization mediated by PKA pathway. **A**, Representative confocal fluorescence images of sensory neurons expressing eGFP-PKC Apl II during a 90 min exposure to 5HT followed by a 45 min wash and then a 5 min 5HT application, all in the presence of anisomycin. **B**, Quantification of PKC Apl II translocation (bars) and modeling output (line). Blue bars are data used from Farah et al. (2009) to fit the model parameters. Red bars are from the present study (n = 10 cells). **C**, Modeling of S dynamics in response to experimental protocol. Black line represents the ratio of S_OFF_ and S_ON_ to total S, the red line the ratio of S_IN2_ to total S, and the blue line represents ratio of S_PKA_ to total S. **D**, Comparing PKC translocation at 135 min after the second 5HT pulse with PKA inactive (anisomycin and KT5720) and PKA active (anisomycin). Student's unpaired two-tailed T test conducted and statistical significance of p<0.01 illustrated by *.

The black and blue networks (Figure 3A) make use of the previously described PKA activity model to affect the desensitization of PKC translocation. Two data sets were used to estimate the parameters of the blue component of the model: one continuous 90 min application of 5HT in the presence of anisomycin and five pulses of 5HT each lasting 5 min with 15 min washes in between, all in the presence of anisomycin [13]. The parameters were estimated to fit both data sets. The conversion of S_OFF_ into S_PKA_ is modeled using mass action kinetics. The recycling of S_PKA_ back into S_OFF_ is inhibited by PKA and is modeled using a combination of mass action kinetics and an inhibitory Hill function (see Materials and Methods section). This network architecture resulted in an excellent fit to both data sets (R^2^ = 0.99 for massed training and 0.88 for spaced training) (Figures 4D, 5B). It was not possible to replicate both the massed training and spaced training data sets without including the PKA inhibition of S_PKA_ recycling. Without this inhibition, fitting the massed training data set caused too much desensitization during spaced training and fitting the spaced training data set caused insufficient desensitization during massed training.

Massed training in the absence of protein synthesis leads to more desensitization of PKC Apl II translocation when PKA is active [13]. However, the model predicts that soon after 5HT is washed away, PKA becomes inactive and S_PKA_ can recycle back to S_OFF_. This recycling suggests that unlike S_IN2_ mediated desensitization, PKA induced desensitization recovers quickly. Thus, when we simulate a 90 min exposure to 5HT followed by a 45 min wash and then a 5 min pulse of 5HT (as above, but in the absence of a PKA inhibitor), the model predicts a considerable recovery of PKC translocation (Figure 5B, C). This recovery happens because during the 90 min stimulation, the majority of S is held in S_PKA_, and during the wash most of S_PKA_ recycles back to S_OFF_. This recycling allows for a greater amount of PKC translocation compared to when PKA was inhibited and the majority of S is found in S_IN1_ (Figure 3C). To test this prediction of the model, we conducted a new imaging experiment, measuring the translocation of eGFP-PKC Apl II during the application of the above protocol (Figure 5A). The translocation of PKC Apl II caused by the 5 min pulse of 5HT after the 45 min wash is in agreement with the modeling prediction, thus validating this component of the model (Figure 5B). The amount of desensitization of PKC Apl II translocation during the massed training is equivalent to that observed by Farah et al. (2009) and, as in that study, PKA increases the amount of desensitization during massed training. However, despite this increased desensitization in the presence of PKA, active PKA increases the recovery from desensitization, as predicted by the model. The large difference between the recovery in the presence or absence of the PKA inhibitor, KT5720, is illustrated in Figure 5D.

### Rescue from desensitization by Anti-Desensitizer (AD) protein

**Figure 6:**
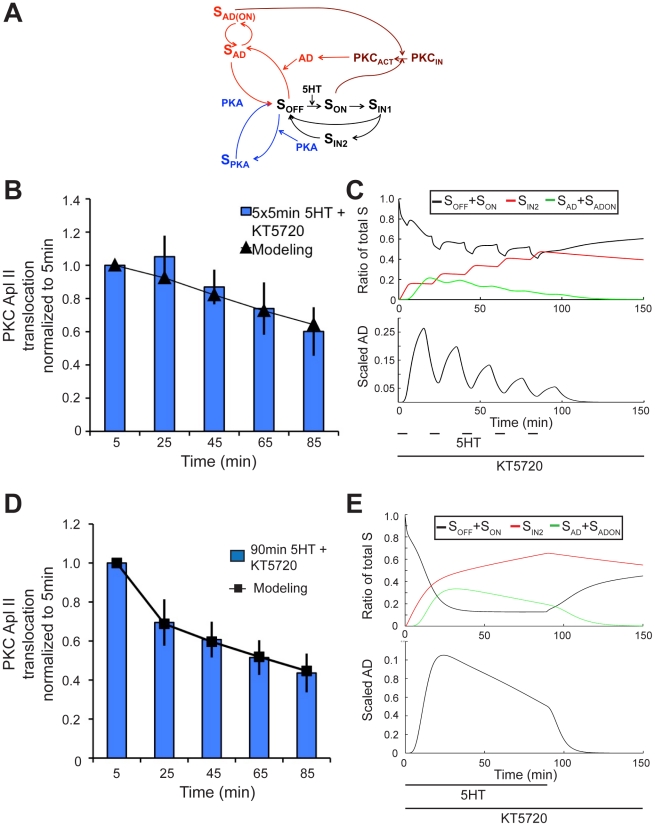
Modeling the rescue from desensitization by Anti-Desensitizer (AD) protein. **A**, Model network pathways of AD desensitization protection pathway (red network designates AD pathway). **B**, PKC Apl II translocation in response to 5×5 min application of 5HT with 15 min washes in between and KT5720 present throughout from Farah et al. (2009) (bars) and modeling output (line). **C**, Top panel represents S dynamics in response to experimental protocol from **B**. Black line represents the ratio of S_OFF_ and S_ON_ to total S and the red line the ratio of S_IN2_ to total S, and green line represents the ratio of S_AD_ and S_ADON_ to total S. Bottom panel represents the amount of AD over time. **D**, PKC Apl II translocation in response to 90 min application of 5HT and KT5720 present throughout from Farah et al. (2009) (bars) and modeling output (line). **E**, Top panel represents S dynamics in response to experimental protocol from **D**. Line colours similar to in **C**.

The rescue from desensitization by the AD protein is modeled using the black and red network components in combination (Figure 6A, red pathway). Two data sets were used to estimate the parameters of this component of the model: 90 min application of 5HT in the presence of KT5720 and five 5 min pulses of 5HT with 15 min washes in between, all in the presence of KT5720 [13]. The model produced an excellent fit to both data sets (R^2^ = 0.95 for spaced training and 0.99 for massed training) (Figure 6B, D). One of the unexpected predictions of the model was both a fast degradation of AD, (with a half-life of ∼5 min) and a slow rate of the S_AD_→S_OFF_ recycling.

**Figure 7:**
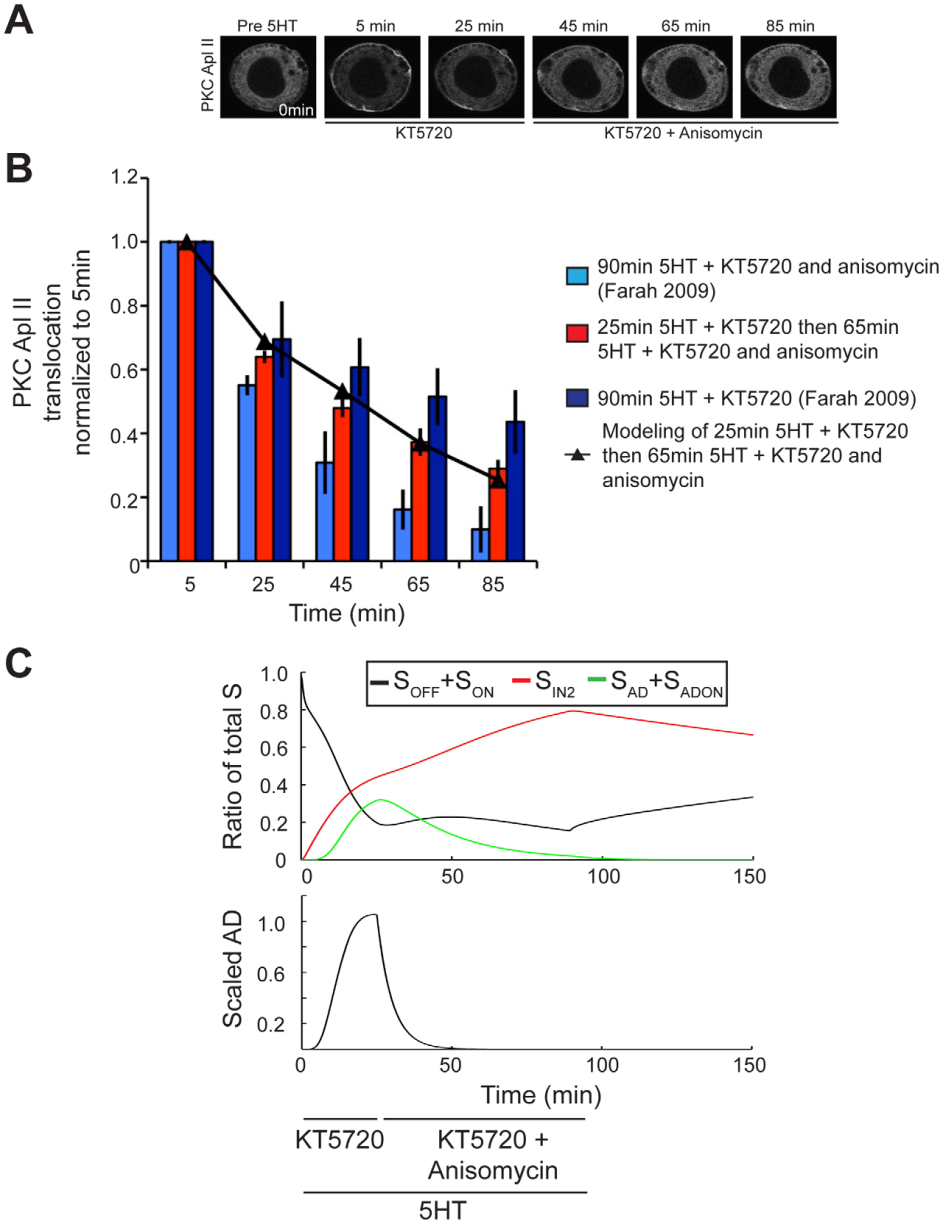
Experimental validation of AD dynamics. **A**, Representative confocal fluorescence images of sensory neurons expressing eGFP-PKC Apl II during a 90 min exposure to 5HT where during the first 25 min KT5720 alone was applied, while during the last 40 min both anisomycin and KT5720 was applied. **B**, Quantification of PKC Apl II translocation (bars) and modeling output (line with square points). 90 min 5HT with KT5720 and anisomycin (light blue bars) is similar to that shown in Figure 1C and 90 min 5HT with anisomycin (dark blue bars) is similar to that shown in Figure 4D. These data points, from Farah et al. (2009), are reproduced here for comparison purposes with the following newly acquired data. Error bars are SEM. Red bars represent quantification of eGFP-PKC Apl II translocation during experimental protocol from **A** (n = 9 cells) compared to line with triangles for the modeling prediction of this experimental protocol (25 min 5HT with KT5720 followed by 65 min with KT5720 and ansiomycin). **F**, Top panel represents S dynamics in response to experimental protocol from **D**. Black line represents the ratio of S_OFF_ and S_ON_ to total S and the red line the ratio of S_IN2_ to total S, and green line represents the ratio of S_AD_ and S_ADON_ to total S. Bottom panel represents the amount of AD over time.

To validate this component of the model, we designed a protocol that would be sensitive to the fast degradation rate of AD. This protocol consisted of exposure to 25 min of 5HT in the presence of KT5720 then 65 min of 5HT in the presence of both KT5720 and anisomycin, with no wash in between. This protocol allows for the indirect observation of the degradation of AD and the recycling of S_AD_ back into S_OFF_. The addition of anisomycin will terminate the translation of AD. During these last 65 min, the model predicts that AD will decay and thus be less effective at transforming S_OFF_ into S_AD_ (Figure 6C). The model further predicts that the absence of AD will cause the remaining S_AD_ to recycle back into S_OFF_, where it will lose its protection from the homologous desensitization pathway, which will manifest in decreased PKC Apl II translocation. Thus, by observing the increased amount of desensitization of this protocol in comparison to when AD translation is present throughout, we can validate the model's predicted rate of AD degradation and rate of S_AD_ recycling back into S_OFF_. To test these predictions of the model, a new imaging experiment was performed by applying this protocol to *Aplysia* sensory neurons expressing eGFP-PKC Apl II. As expected, the amount of PKC translocation observed in these neurons during the first 25 min of 5HT was equivalent to that observed during the 25 min of massed training in the presence of KT5720, carried out by Farah et al. (2009) (Figure 6A, B). However, the final 65 min of this protocol, where both KT5720 and anisomycin are present, caused a lower amount of PKC Apl II translocation compared to that caused by massed training in the presence of only KT5720, in agreement with the model prediction (R^2^ = 0.99) confirming the fast degradation rate of AD and the slow rate of S_AD_ to S_OFF_ (Figure 7C).

### Modeling increase in desensitization by Desensitizer (D) protein

**Figure 8:**
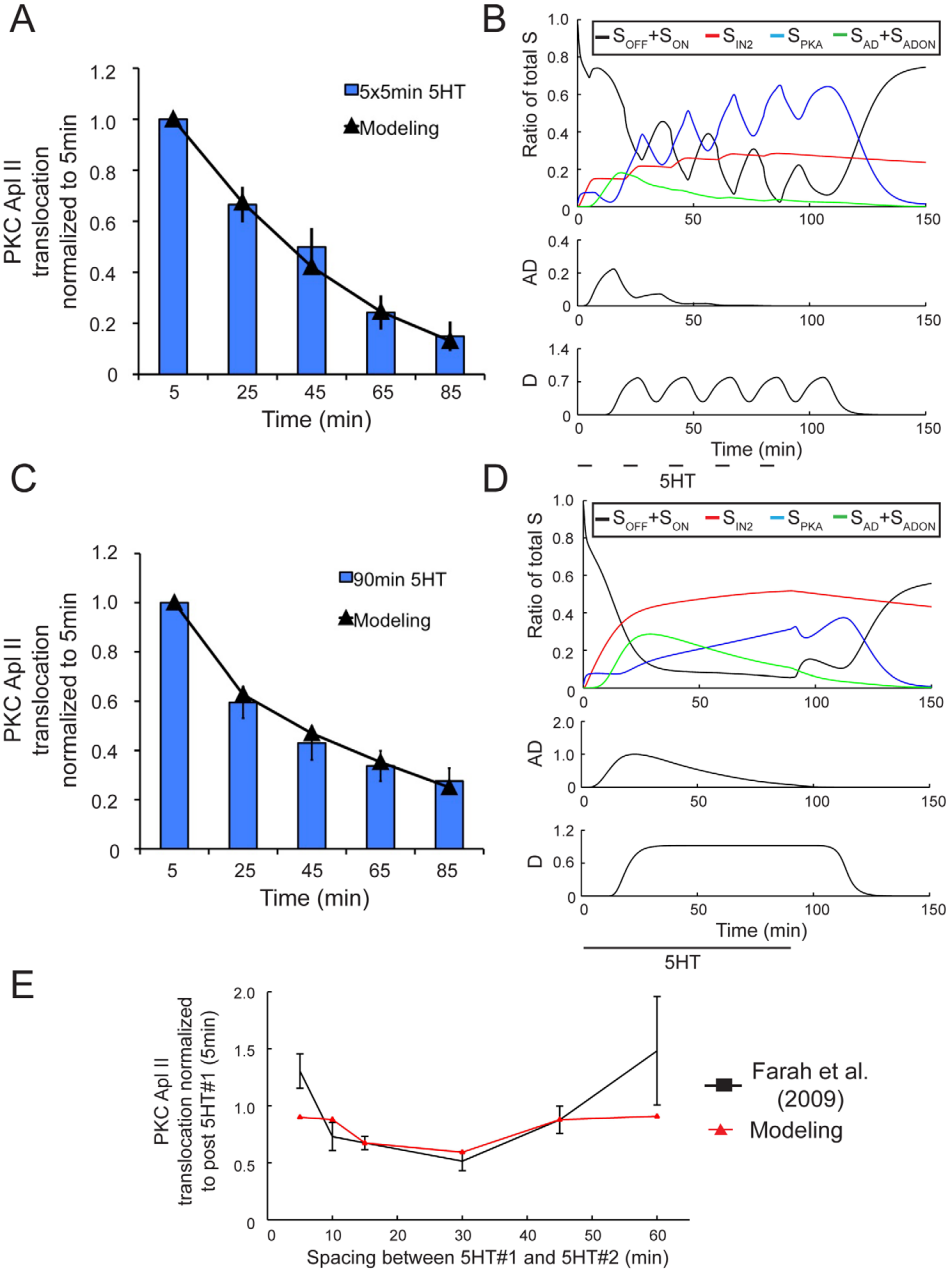
Fitting complete model to PKC translocation measured during no pharmacological interventions. **A**, PKC Apl II translocation in response to 5×5 min application of 5HT with 15 min washes in between (bars) and modeling output (line). **B**, Top panel represents S dynamics in response to experimental protocol from **A**. Black line represents the ratio of S_OFF_ and S_ON_ to total S, red line the ratio of S_IN2_ to total S, green line represents the ratio of S_AD_ and S_ADON_ to total S, and blue line represents the ratio of S_AD_ and S_ADON_ to total S. Middle panel is the amount of AD over time and the bottom panel is the amount of D over time. **C**, PKC Apl II translocation in response to a 90 min application of 5HT (bars) and modeling output (line). **D**, Top panel represents S dynamics in response to experimental protocol from **G**, with line colours identical to in **B**. Middle panel represents the amount of AD over time and the bottom panel the amount of D over time. **E**, PKC Apl II translocation in response to 2×5 min applications of 5HT with varying wash periods in between (black line) and modeling output (red line).

During spaced training, the desensitization of PKC Apl II translocation was increased in control cells in comparison to when protein translation was inhibited. This increase in desensitization was observable only when both PKA activity and protein translation are allowed to proceed, meaning a translated protein is mediating this increase and its rate of translation is dependent on PKA activity. We name this hypothetical protein Desensitizer (D), and its effects on PKC Apl II translocation are modeled by the green component of the network (Figure 2). Seven data sets were used to estimate the parameters of this component of the model: one continuous 90 min application of 5HT, five pulses of 5HT each lasting 5 min with 15 min washes in between, and five experiments, each with two pulses of 5HT each lasting 5 min but with a different wash period length (5 min, 10 min, 15 min, 30 min, 45 min,) in between the pulses [13] (Figure 8A, C, E). The resulting model formed an excellent fit to the data (R^2^ = 0.99 for massed training, 0.99 for spaced training, and 0.75 for two pulses of 5HT with varying wash intervals). One exception is the 5 min pulse followed by a 5 min wash, where there is an increase in PKC Apl II translocation compared to the initial translocation, while our model shows no increase in translocation. We believe fitting this increase would require a more detailed dissection of the pathway between the GPCR and its downstream targets and is beyond the scope of this study.

### Model successfully predicts the response to new spacing protocols

**Figure 9:**
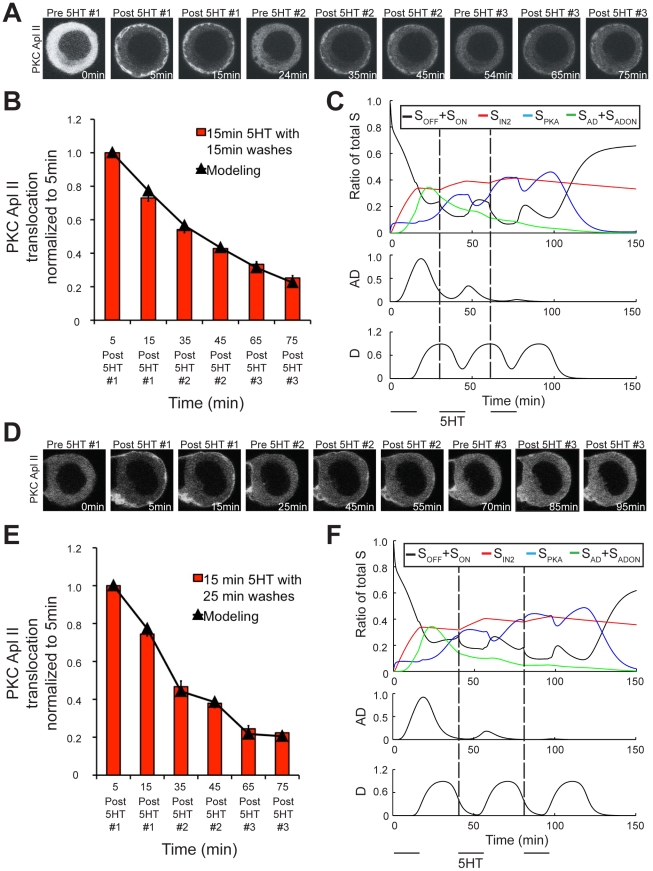
Longer wash periods lead to greater desensitization of PKC Apl II translocation. **A**, Representative confocal fluorescence images of sensory neurons expressing eGFP-PKC Apl II during a 3×15 min application of 5HT with 15 min washes in between. **B**, Quantification of PKC Apl II translocation (bars, n = 9 cells) and modeling output (line). Error bars are SEM. **C**, Top panel represents S dynamics in response to experimental protocol from **A**. Black line represents the ratio of S_OFF_ and S_ON_ to total S, red line the ratio of S_IN2_ to total S, green line represents the ratio of S_AD_ and S_ADON_ to total S. Middle panel represents the amount of AD over time and the bottom panel the amount of D over time. Dotted line represents time of second and third 5HT application. **D**, Representative confocal fluorescence images of sensory neurons expressing eGFP-PKC Apl II during a 3×15 min applications of 5HT with 25 min washes in between. **E**, Quantification of PKC translocation (bars, n = 6 cells) and modeling output (line). Error bars are SEM. **F**, Top panel represents S dynamics in response to experimental protocol from **E**. Black line represents the ratio of S_OFF_ and S_ON_ to total S, red line the ratio of S_IN2_ to total S, green line represents the ratio of S_AD_ and S_ADON_ to total S. Dotted line represents time of second and third 5HT application. Middle panel represents the amount of AD over time and the bottom panel the amount of D over time.

**Figure 10:**
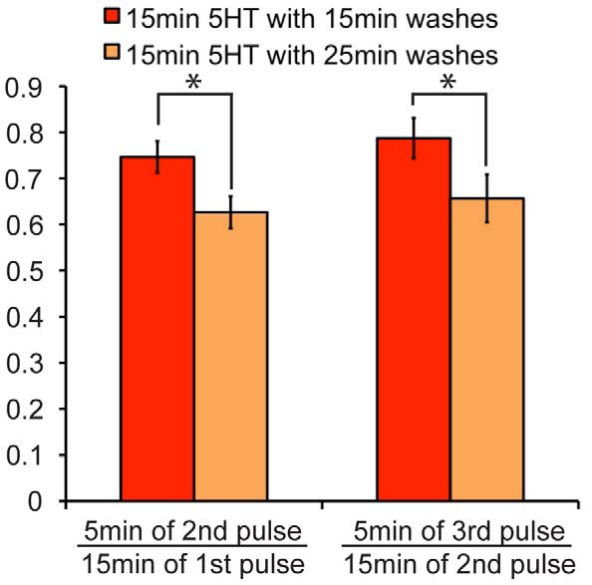
Quantifying the amount of desensitization of PKC Apl II translocation occurring during wash periods. Left pair of bars represent the amount of PKC Apl II translocation at the 5 min point of the second pulse divided by the amount of PKC Apl II translocation at the 15 min point of the first pulse. Right pair of bars represents the amount of PKC Apl II translocation at the 5 min point of the third pulse divided by the amount of PKC Apl II translocation at the 15 min point of the second pulse. Red bars correspond to 15 min 5HT with 15 min washes (n = 9) and Orange to 15 min 5HT with 25 min washes (n = 6). Student's unpaired two-tailed T test conducted and statistical significance of p<0.05 illustrated by *.

As one of the rationales for generating this model was to gain insight into the role of spacing, our final confirmation of the model tested an alternate spacing protocol. We designed an experiment that would require the functioning of all the model components and that made a specific prediction that was not obvious and could be tested. Interestingly, we found that if 15 min pulses of 5HT were used, the model predicted that longer washes would lead to increased desensitization. While 15 min pulses produce both D and AD, the model predicts that longer washes will reduce the levels of AD compared to D and thus predicts greater desensitization by longer washes (Figure 9B and E). In particular note that the model predicts that with the shorter spacing (Figure 9C), the amount of S complex in S_AD_ is larger than in S_PKA_ immediately before the second pulse, while with longer spacing (Figure 9F), the model predicts that there is more S complex in S_PKA_, than in S_AD_. Thus, the second pulse of serotonin during the protocol with longer spacing should be less able to translocate PKC Apl II because of the conversion of S_AD_ to S_PKA_. To test this prediction, we performed a new imaging experiment where sensory neurons were exposed to three 15 min pulses of 5HT with either 15 min or 25 min washes in between the 5HT pulses. The results of this protocol are also sensitive to the delay and rate of D translation (parameters that had not yet been validated in a separate experiment). Both protocols were applied to *Aplysia* sensory neurons expressing eGFP-PKC Apl II. The amount of PKC translocation during both protocols matched the modeling prediction (R^2^>0.99) (Figure 9A, B, D, and E) and thus validates this component of the model as well as the functioning of the completed model. In particular, to highlight the effect of the wash, we calculated the amount of desensitization during the 15 min or 25 min wash (e.g. the amount of translocation at the beginning of pulse 2 compared to the end of pulse 1, or the beginning of pulse 3 compared to the end of pulse 2). The model predicted more desensitization during the longer wash and this was confirmed by the imaging experiment (Figure 10).

### Sensitivity analysis

A parameter sensitivity analysis was performed on the completed model to investigate which parameters were most important in driving the results of the model. Each parameter was varied between +/−5% and +/−50% while holding the other parameters at their defined values. The model was then simulated using a 90 min application of 5HT and its resulting PKC Apl II translocation compared to that observed by Farah et al. (2009), which was initially used to fit the model. The sensitivity of a parameter was classified as High if either a +/−5% change in its value caused a change in the fit of the data of over 25%. Similarly, the sensitivity of a parameter was Medium if either a +/−50% change in value caused a change in the fit of the data of over 25%, and Low if the +/−50% change in value did not change the fit by more than 25%. This was then repeated using a spaced application of 5HT (5×5 min 5HT with 15 min washes). The complete sensitivity analysis is summarized in Table 1.

Of the 41 parameters, 5 were classified as High, 12 as Medium, and 6 as Low in both massed and spaced training sensitivity analysis. Interestingly, the majority of the parameters (3/5) with high sensitivity for both types of training were those associated with the initial component of the model responsible for activating PKC Apl II. The remaining two parameters involved how AD works (the synthesis rate and its ability to stop S from going into S_PKA_). It is not surprising that changing parameters that affect the initial translocation of PKC Apl II by 5HT and its decay after 5HT is removed would have a large effect on the model output, since the model was built around this core. However, these parameters were chosen in a somewhat arbitrary fashion to fit the initial data since the actual rates of DAG synthesis and decay are not known in this system. To ensure that the set of values we chose for these parameters are not critical for the working of the model, we found another parameter set that could fit the initial translocation data (see Table 1). Reassuringly, the rest of the model still worked, suggesting that the model was not dependent on the actual values for these initial parameters, just the ability of the model to replicate the known rate of PKC Apl II translocation and dissociation by 5HT.

The remaining 18 parameters had sensitivities dependent on the type of 5HT application profile. Interestingly, about the same number of parameters had specific high sensitivity for massed (5) vs spaced (4). For spaced, two of these are again from the initial model and the others concern the synthesis rate of D and AD. Similarly, for massed, two of these are for the initial model and the others concern the synthesis rate for D. The sensitivity analysis suggests, similar to the experiments, that the critical parameters that determine the model are involved in the synthesis of D and AD.

## Discussion

We have successfully modeled the differences in desensitization of the PKC Apl II response to spaced and massed applications of serotonin. Two major insights were achieved by this modeling. The first was that despite the greater desensitization present with PKA activity present, PKA activity actually protected the ability of 5HT to translocate PKC Apl II at later times due to placing the signaling complex into a state where it could recover. The second insight is that the major determinant of how the system responds to spacing is the production and degradation rate of proteins, suggesting that the production of proteins with short half-lives is a powerful mechanism for cells to be able to sense a time domain in the order of minutes to hours. While this model is probably not unique, its proven ability to predict the results of new experiments suggests that it has captured the essential elements of the biological process it models.

### Biological plausibility of the model

The architecture of our model is based on the fact that desensitization and resensitization of GPCRs is due to endocytosis followed by exocytosis, the simplest biological substantiation of the states of S are distinct endocytic recycling pathways of the receptor or distinct states of the receptor after association with GPCR binding-proteins [29], [39]. Below we review these processes and the plausibility of the parameters we have assigned to these steps. We also review other aspects of the model and whether the parameters and architecture are biologically plausible.

#### S_IN1_, S_IN2_

S_IN1_ would represent a fast recycling pool, whereas S_IN2_ would represent sorting to lysosomes or other slowly cycling pools (i.e. back to the trans-Golgi network) [40], [41]. The rate of S_ON_ to S_IN1_ is less than a minute and similar rates have been seen for internalization of GPCRs using live imaging, suggesting all the steps in this process, including phosphorylation, arrestin binding and endocytosis can occur in less than one minute [22], [42]. The back rate S_IN1_ to S_OFF_ is also very fast, less than one minute and this is faster than most measurements for resensitization of GPCRs, where this usually takes minutes [22]; however there are fast recycling pathways from endosomes that do occur in this time range and thus this transition is still plausible [43]. It should also be noted that both S_ON_ to S_IN1_ and S_IN1_ to S_OFF_ rates are part of the initial parameter set, for which we have already found multiple parameter sets that could match the data without changing the overall functioning of the model. S_IN1_ to S_IN2_ has a rate of minutes, which is reasonable for sorting into a late endosome [22], [24]. It should be noted that there are multiple steps after the initial sorting event prior to degradation in lysosomes, and actual degradation usually is not seen until hours after internalization [22], [24]. The very slow return rate from S_IN2_ is consistent either with slow recycling or degradation [22], [24] and we would need to examine much longer recovery times to resolve this issue.

#### S_PKA_

S_PKA_ would represent localization to a regulated recycling pool [24]. The rate of heterologous desensitization (S_OFF_ to S_PKA_ - minutes) is consistent with rates observed for heterologous desensitization of other GPCRs [44]. It is known that phosphorylation of receptors or associated proteins can regulate the pool that they are found in [39] and thus the ability of PKA phosphorylation to put the signaling complex into a specific endocytic pool is plausible. Why PKA activity is required for inhibition of the recycling pathway is less obvious. One could envision a receptor binding protein important for recycling whose ability to bind is blocked by continuous PKA phosphorylation. Alternatively, PKA may regulate vesicular trafficking proteins that regulate the fusion of a storage vesicle with the membrane.

#### S_AD_

The AD protein must prevent inactivation, prevent endocytosis and promote faster recycling. It presumably does this by binding to the GPCR. This binding can occur either to S_OFF_ or S_PKA_ and in either case transforms S into S_AD_. In the model, the requirement for the AD protein to recover the signaling complex from S_PKA_ suggests that the binding is dominant over the PKA site and may be equivalent to the increased recycling seen when PKA is turned off. Presumably the binding of AD would compete with the speculated receptor binding protein important for retaining the signaling complex in a PKA sensitive pool as well as prevent cycling into S_IN2_. A number of proteins have been identified that can both retain GPCRs on the membrane and speed recycling of GPCRs, mostly PDZ proteins that bind to the carboxy-terminus of receptors, and these would be candidates for AD [39], [45]. Interestingly PDZ proteins such as PSD-95 have been shown to be targets of fast synthesis and degradation [46], [47].

Our model makes strong predictions concerning AD that will be useful to ascertain the true identity of the AD protein. We predict that over expression of the protein would be sufficient to prevent desensitization. Furthermore, we predict that the protein should bind directly to the GPCR that is responsible for PKC activation. In the future, it will be interesting to test orthologues of these PDZ proteins in *Aplysia* to identify binding partners to the GPCR that have these properties.

The slow rate of S_AD_ to S_OFF_ is surprising, as one would expect this rate to be fast once AD is degraded. It is possible that the AD bound to the receptor degrades more slowly than the free pool of AD. If AD was a PDZ domain-containing protein, the off rate of PDZ proteins can be quite slow [48]. It should also be noted that the S_PKA_ to S_AD_ rate is slower than the S_OFF_ to S_AD_ rate. Since both rates may involve binding of AD to the receptor, one might expect them to be the same. However, since S_OFF_ and S_PKA_ may represent separate compartments, the availability of AD could be different. It is also possible that AD is slower to bind the phosphorylated receptor than the non-phosphorylated receptor.

#### D

The D protein promotes the movement of the complex into the regulated recycling compartment. One protein that would be a candidate for this would be the clathrin light chain that is known to be translationally produced downstream of PKA in sensory neurons [49] and is associated with target-specific clathrin-mediated endocytosis events [50], [51]. Since D mainly increases the ability of PKA to move S into S_PKA_, it would be consistent for D to be PKA itself or a protein involved in increasing activation of PKA. However, while there is a protein-synthesis dependent activation of PKA after spaced training [10], this is not observed until after 4 or 5 pulses of 5HT, while D has a major effect after a single pulse of 5HT. The amount of PKA activation is not different after the second or third pulse compared to the first pulse [10].

#### Speed of protein synthesis and degradation

The model predicts that proteins are produced and degraded quite quickly, in a time scale of minutes. The fast rate of protein production for proteins involved in plasticity is becoming more widely accepted. In mGLUR-LTD in hippocampal neurons, levels of Arc are significantly increased 5 min after treatment [52]. In *Aplysia*, protein synthesis is required for plasticity after 5HT as soon as 5 minutes after training or 5HT application [53], [54]. The degradation rates of the proteins are also fast, but not inconsistent with other plasticity related proteins such as Arc whose half-life appears to be on the order of minutes [55], [56]. The delay in production of D could be biologically caused by a number of different models. One possibility is in the delayed activation of the translation factors required for initiation. A larger protein will have a longer lag between initiation and production of protein. There may be a cascade of factors involved and perhaps translation of an additional protein is required to initiate translation of D. While the model puts the lag in the production of D, it may be that after production D requires time to act, either due to a requirement for posttranslational modification, or transport to an important site. This would lead to an equivalent delay.

#### Integrals, thresholds, and Hill functions

It was important to allow AD protein to be synthesized during massed, but not spaced, applications of 5HT, as discussed above. This required us to differentiate the amount of time that PKC Apl II was activated (longer in massed, than in spaced). We accomplished this by integrating PKC Apl II activity over time and then applying a Hill function in order to generate a threshold of PKC Apl II activity that would lead to AD synthesis. We also used Hill functions in a number of other steps (D synthesis and transforming or inhibiting the transformation of S into its different states) as they are well-behaved mathematical functions that are widely used in biological modeling, especially when a nonlinear saturating dynamic is desired and there is limited biological information on the actual mechanisms underlying step or when multiple reactions are modeled in one step [57].

### Comparison to other models that explain timing during plasticity

Spaced vs. massed training occurs in a number of different time scales, thus molecular mechanisms are required that act in different time domains. For example, induction of LTP by spaced stimuli requires PKA, but not when massed stimulation is used [58], and the spaced stimuli were required to recruit protein synthesis-dependent mechanism [59]. A recent model explains this finding based on the differential effects of calcium on PKA and CAMKII. This model depends on inter-trial intervals that range between seconds and 5 minutes [60]. The frequency dependent activation of CAMKII is sensitive to timing intervals in this period and is proposed to be the mechanism for sensing the spacing between stimuli [61]. In mice object recognition was enhanced by spacing of 15 min, compared to 5 min or massed training [62]. In mice that lacked Protein Phosphatase 1 (PP1), 5 min spacing was sufficient for learning. In this case the rate-limiting step for learning was activation of CREB, and spacing was required in order for PP1 to be deactivated before the next training trial allowing for CREB activation [62]. CREB activation is also the proposed difference between spaced and massed learning in *Drosophila* odor avoidance [63]. In *Drosophila*, spacing is regulated by waves of MAP kinase activation where both the activation and decay kinetics appear critical for the spacing interval [64]. In *Aplysia*, it has recently been demonstrated that for long-term facilitation, only two spaced trials are required, 45 minutes apart, but neither 30 nor 60 minute spacing is adequate [65]. Again, in this case the spacing corresponds to a wave of MAP kinase activation [65]. None of these cases directly implicate the rates of protein synthesis or degradation as critical for timing, although it is possible that the induction of MAP kinase activation at later times may require protein synthesis. Interestingly, the activation of CREB in mammals appears to require removal of CREB repressor, which requires both blockade of translation through eIF2a dephosphorylation [66] and increased degradation due to proteosomal activation [67]. Thus, in this case regulating the level of a protein also may mediate differences between spaced and massed trained determining whether or not transcription is activated. It will be interesting in the future to determine how generally neurons sense time through measuring the half-life of newly synthesized proteins.

## Materials and Methods

### Mathematical modeling

A mathematical model of the desensitization of PKC Apl II translocation in *Aplysia californica* sensory neurons was constructed in the MATLAB programming environment. The model consists of a system of integro-differential equations with delays, where each equation describes the change in concentration of the proteins PKC Apl II, PKA, Desensitizer (D), Anti-Desensitizer (AD), and of each instance of the signaling complex (S). Since we are only interested in PKC Apl II translocation occurring between the cytosol and plasma membrane [18] a single compartment model was used.

The complete model is depicted in Figure 2. The colours of this figure correspond to the components of the model. The model was constructed in a sequential manner. First, the components outlined in black and maroon were fit to data [13] at which point its parameters were specified and not allowed to change. Following these component's completion, the component outlined in blue was similarly constructed, then the red component and finally the green component. In order to illustrate this sequential construction within the model equations, we have named the parameters according to which component they reside in: A for the black component, B for blue, C for red, and D for green.

The most basic component of the model is the translocation of PKC Apl II from the cytosol to the plasma membrane (maroon component). This translocation is proportional to the concentration of diacylglycerol (DAG) on the membrane and thus the translocation is given by the following equation:
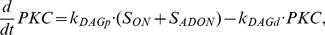
(1)where k_DAGp_ is the rate of PKC Apl II translocation to the membrane, and k_DAGd_ the rate of PKC Apl II removal from the membrane. S_ON_ represents the proportion of S currently in the active state, which is capable of translocating PKC Apl II to the membrane. The inactive state of S is given by S_OFF_, and S_IN1_ is a transition state between S_ON_ and S_OFF_. S can be transformed into 3 other states, as will be described next. We require the total amount of S to remain constant by employing the following restriction: 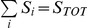, where i = ON, OFF, IN1, IN2, PKA, AD, and D. We scale each S variable by 1/S_TOT_, such that all parameters k_i_, i = A1–A5, B1–B2, C1–C2, and D1–D3 will have units min^−1^. We have set S_TOT_ = 1, where we refrain from assigning units to the S variables since we cannot measure the concentrations of PKA or PKC in *Aplysia* neurons in order to accurately define a unit of measure. Furthermore, units are not assigned to any variable with concentration as a possible dimension. This simplification is justified since we have developed a single compartment model of *Aplysia* sensory neurons to qualitatively describe the dynamics of PKC desensitization. Using non-dimensional variables and parameters allows us to observe important dynamics, such as relative magnitudes of proteins and the time course of S recycling, which allow us to gain insight into the molecular regulatory mechanism involved in the desensitization of PKC translocation.

The following equations describe the rates of change of concentration of the first four S states:
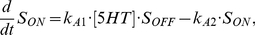
(2)

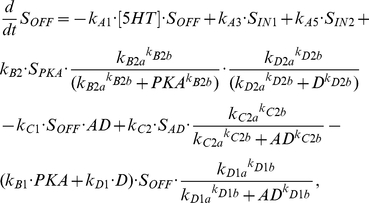
(3)

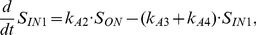
(4)

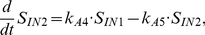
(5)where [5HT] represents the concentration of 5HT being applied to the system and is given a standard value of 10 µM during any application of 5HT, k_A1_ represents the transformation of S_OFF_ into S_ON_, k_A2_ of S_ON_ into S_IN1_, k_A3_ of S_IN1_ into S_OFF_, k_A4_ of S_IN1_ into S_IN2_, and k_A5_ of S_IN2_ into S_OFF_. The additional terms in equation (3) refer to the further transformations that S_OFF_ can undergo. Without these additional terms these equations describe the black model in Figure 3A. The first additional transformation of S_OFF_ is mediated by the catalytic subunit of PKA, where S_OFF_ is converted to S_PKA_, which has the following equation:
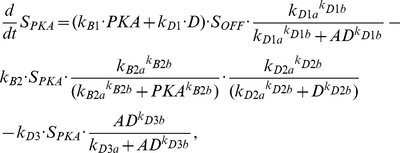
(6)where k_B1_ is rate constant of the transformation from S_OFF_ into S_PKA_, which is brought about by the activity of the catalytic subunit of PKA or the protein D. However, the protein AD, through a Hill function with coefficient k_D1b_ and half saturation k_D1a_, can inhibit this conversion. The recycling of S_PKA_ into S_OFF_ occurs with rate constant k_B2_, but is inhibited by PKA and D through inhibiting Hill functions with coefficients k_B2b_ and k_D2b_, respectively, and half saturations k_B2a_ and k_D2a_, respectively. Also, activity of AD can convert S_PKA_ into S_AD_ with a rate constant k_D3_ and a Hill function with coefficient k_D3b_ and half saturation k_D3a_.

The dynamics of the catalytic and regulatory subunits of PKA are adapted from a model presented by Pettigrew et al. (2005), where the changes to this model are described in the results. PKA dynamics are given in the following equations:
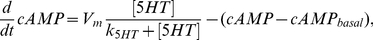
(7)

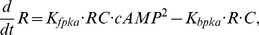
(8)

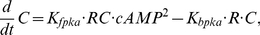
(9)

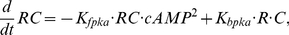
(10)Where V_m_ is the cAMP synthesis rate constant, and K_5HT_ is the half saturation of the Hill function associated with cAMP synthesis. K_fpka_ is the rate constant associated with the dissociation of the catalytic and regulatory subunits, while the reassociation rate is given by K_bpka_. The amount of PKA activity is set equal to the amount of the free catalytic subunit (C); PKA = C. The parameters associated with protein synthesis are given the subscript S to differentiate them from signaling complex dynamics. The synthesis of AD and D are given by the following equations:
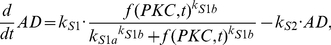
(11)

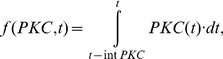
(12)

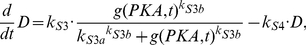
(13)

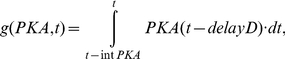
(14)


AD synthesis depends on the total activity of PKC Apl II over a previous time window of duration given by intPKC in equation (12). The integration of PKC Apl II activity leads to the synthesis of AD through a Hill function with coefficient k_S1b_ and half saturation k_S1a_. k_S2_ represents the AD degradation constant. Similarly, D synthesis depends on an integration of PKA activity over a time period of intPKA in equation (14), which leads to the synthesis of D with a rate constant of k_S3_ and through a Hill function with coefficient k_S3b_ and half saturation k_S3a_. The degradation of D is given by rate constant k_S4_. D leads to the transformation of S_OFF_ into S_PKA_, which was described above in equation (6), while AD transforms S_OFF_ into S_AD_, whose dynamics are modeled with the following equation:
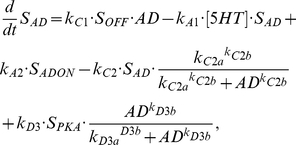
(15)where k_C1_ is the rate constant associated with the transformation of S_OFF_ into S_AD_, k_C2_ the rate constant of the recycling of S_AD_ into S_OFF_, which can be inhibited by AD through a Hill function with coefficient k_C2b_ and half saturation k_C2a_. Also, S_AD_ can become activated when 5HT transforms it into S_ADON_, whose dynamics are given in the following equation:
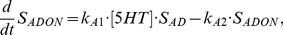
(16)whose transformation and recycling rate constants are identical to those of S_OFF_ into S_ON_. S_ADON_ activates PKC Apl II in an identical fashion to S_ON_ in equation (1).

The system was solved numerically by employing a 4^th^ order Runga-Kutta scheme to solve the differential equations and the Trapezoid Rule to solve the integrals [68]. Parameter estimation was conducted with the help of the MATLAB Optimization Toolbox and Global Optimization Toolbox, specifically the functions lsqcurvefit, ga, and fmincon. These functions were used to minimize the least squares distance between the modeling output and experimental data. Values of individual parameters are given in Table 1.

### Aplysia cell culture preparation

Adult *Aplysia californica* (76 to 100 g; University of Miami *Aplysia* Resource Facility, RSMAS, FL) organisms were anesthetized by an injection of 50 to 100 ml of 400 mM (isotonic) MgCl2. Pleuropedal ganglia were removed and digested in L15 medium containing 1% protease type IX (Sigma). L15 medium was purchased from Sigma and supplemented with 0.2 M NaCl, 26 mM MgSO4·7H2O, 35 mM dextrose, 27 mM MgCl2·6H2O, 4.7 mM KCl, 2 mM NaHCO3, 9.7 mM CaCl2·2H2O, 15 mM HEPES, and the pH was adjusted to 7.4. Following digestion, tail sensory neurons were isolated and plated in L15 medium containing 50% *Aplysia* hemolymph on MatTek glassbottom culture dishes (MatTek Corporation, Ashland, MA) with a glass surface of 14 mm and a coverslip thickness of 0.085 to 0.13 mm. The dishes were pretreated with poly-L-lysine (molecular weight, >300,000; Sigma).

### Plasmid construction and microinjection of plasmid vectors

The pNEX3 enhanced green fluorescent protein (eGFP) PKC Apl II has been described previously [13], [19], [69]. On day 1 after isolation, solutions of plasmids in distilled water containing 0.25% fast green were microinjected into neurons from back-filled glass micropipettes. The tip of the micropipette was inserted into the cell nucleus, and short pressure pulses (10–50 ms duration; 20 lb/in2) were delivered until the nucleus became uniformly green. The cells were incubated for 4–5 hrs at room temperature and then kept at 4°C until use.

### Confocal microscopy of Aplysia neurons

Neurons expressing eGFP-PKC Apl II were imaged on a Zeiss laser-scanning microscope (Zeiss, Oberkochen, Germany) with an Axiovert 200 and a ×40 or ×63 oil immersion objective with a 25-mW argon laser with 25% laser output. The laser line was attenuated to 4% transmission output prior to live imaging. 5HT (10 µM) was added to the dish in L15 medium containing 50% hemolymph. 5HT was washed away with artificial seawater (ASW; 10 mM HEPES, pH 7.5, 0.46 M NaCl, 10 mM KCl, 11.2 mM CaCl2·2H2O, 55 mM MgCl2·6H2O). For spaced training, neurons received five applications of 10 µM 5HT (5 min each) at an intertrial interval (ITI) of 20 min. For massed training, neurons received a single continuous application of 10 µM 5HT for 90 min. All experiments were performed at room temperature (20 to 23°C).

### Drug treatment

Anisomycin (Sigma-Aldrich), and KT5720 (Calbiochem) were used at concentrations of 50 µM, and were present in the media throughout spaced or massed training. There was no pre-incubation with these drugs prior to 5HT treatment and because of this we erred on the high side of the concentrations that have been used previously. The controls used in all experiments were always performed from the same batch of animals when the drugs were used. The translation inhibitor anisomycin was purchased from Sigma-Aldrich.

### Image analysis

The level of PKC Apl II translocation for each cell was determined by tracing three rectangles at random locations at the plasma membrane and three rectangles at random locations in the cytosol. The width of the membrane rectangles was three-five pixels wide to avoid cytoplasmic contamination, but otherwise the size of the rectangles was not constrained. The average intensity at the membrane (Im) and the average intensity in the cytosol (Ic) was then calculated and the Im/Ic ratio is the degree of membrane association. In all figures, the control used to normalize the translocations in the presence of a drug is the post 5HT #1 in the presence of that drug.
